# Adrenomedullin blockade suppresses sunitinib-resistant renal cell carcinoma growth by targeting the ERK/MAPK pathway

**DOI:** 10.18632/oncotarget.11463

**Published:** 2016-08-22

**Authors:** Yongqian Gao, Jinyi Li, Na Qiao, Qingsong Meng, Ming Zhang, Xin Wang, Jianghua Jia, Shuwen Yang, Changbao Qu, Wei Li, Dongbin Wang

**Affiliations:** ^1^ Department of Interventional Radiology, Tangshan Gongren Hospital, Hebei Medical University, Tangshan 063000, P.R. China; ^2^ Department of Urology, Icahn School of Medicine at Mount Sinai, New York, 10029, USA; ^3^ Department of Urologic Surgery, The Second Hospital of Hebei Medical University, Shijiazhuang, 050000, China

**Keywords:** renal cell carcinoma, adrenomedullin, tyrosine kinase inhibitor, ERK

## Abstract

**Purpose:**

To evaluate the mechanisms underlying sunitinib resistance in RCC and to identify targets that may be used to overcome this resistance.

**Results:**

Reanalysis of transcriptome microarray datasets (GSE64052 and GSE76068) showed that adrenomedullin expression was increased in sunitinib-resistant tumors. And adrenomedullin expression was increased in sunitinib-resistant tumor xenografts, accompanied by upregulation of phospho-ERK levels. However, blocking adrenomedullin inhibited sunitinib-resistant tumor growth. Treatment of RCC cells with sunitinib and ADM22-52 was superior to monotherapy with either agent. Additionally, adrenomedullin upregulated cAMP and activated the ERK/MAPK pathway, promoting cell proliferation, while knockdown of adrenomedullin inhibited RCC cell growth and invasion *in vitro*.

**Materials and methods:**

We searched the Gene Expression Omnibus (GEO) database to find data regarding sunitinib-resistant RCC. These data were subsequently reanalyzed to identify targets that contribute to sunitinib resistance, and adrenomedullin upregulation was found to mediate sunitinib resistance in RCC. Then, we created an RCC mouse xenograft model. Mice were treated with sunitinib, an adrenomedullin receptor antagonist (ADM22-52), a MEK inhibitor (PD98059) and different combinations of these three drugs to investigate their effects on tumor growth. RCC cells (786-0) were cultured *in vitro* and treated with an ADM22-52 or PD98059 to determine whether adrenomedullin activates the ERK/MAPK pathway. Adrenomedullin was knocked down in 786-0 cells via siRNA, and the effects of this knockdown on cell were subsequently investigated.

**Conclusions:**

Adrenomedullin plays an important role in RCC resistance to sunitinib treatment. The combination of sunitinib and an adrenomedullin receptor antagonist may result in better outcomes in advanced RCC patients.

## INTRODUCTION

Renal cell carcinoma (RCC) accounts for approximately 2% of adult malignancies worldwide, an incidence that is increasing by 1.5–5.9% per year [1]. The most common histological type (70%–80%) is clear cell RCC (ccRCC). Due to resistance to chemotherapy and radiotherapy, the prognosis of advanced RCC is very poor. However, RCC tends to be highly vascular; therefore, in recent years, angiogenesis inhibitors, including sorafenib and sunitinib, have been used to treat RCC. Both of these agents are receptor tyrosine kinase (RTK) inhibitors and have already been approved for advanced RCC treatment by the FDA [2]. Clinical trial data indicate that both sorafenib and sunitinib are able to improve progression-free survival and overall survival in RCC patients by targeting the vascular endothelial growth factor receptor (VEGFR) and platelet-derived growth factor receptor (PDGFR) [3, 4].

However, the outcomes of anti-angiogenesis therapy in RCC are still unsatisfactory due to the development of resistance during therapy [5, 6], which results in cancer progression. The mechanisms underlying this resistance are not fully understood. It is generally acknowledged that resistance develops due to genetic alterations resulting in the activation of other pathways to compensate for the blockade of the VEGFR [7, 8]. In a study on the survival of RCC patients [9], sunitinib resistance developed during the course of treatment, likely as a result of changes in the tumor microenvironment or gene expression, which facilitated continued tumor growth independently of the VEGFR [10]. Huang D et al. found that IL-8 levels were increased in sunitinib-resistant tumors in which the VEGFR pathway was bypassed, and angiogenesis was promoted [11]. Casanovas O et al. [12] suggested that resistant tumors were able to evade sunitinib treatment due to the activities of multiple fibroblast growth factors (FGFs), which facilitate resumption of tumor angiogenesis and tumor growth. Most of the previous studies on sunitinib-resistant RCC have focused on the identification of compensatory pathways that promote angiogenesis. However, the findings of these studies cannot fully explain the mechanisms underlying the evasion of VEGFR inhibitor treatment in RCC, because some studies have found that continued growth of sunitinib-resistant tumors occurred independently of tumor microvessel density (MVD) [13]. Therefore, a different intracellular pathway that does not mediate angiogenesis likely facilitates sunitinib-resistant tumor growth.

Adrenomedullin (ADM) was first isolated in 1993 from human pheochromocytoma extracts [14]. It is a protein belonging to the amylin/calcitonin gene-related peptide (CGRP) superfamily [15]. The ADM gene is located on chromosome 11p15.4 and encodes a protein comprising 52 amino acids. Elevated ADM levels have recently been observed in a variety of cancers, including RCC, prostate cancer, non-small cell lung carcinoma, and ovarian carcinoma [16]. Fujita Y et al. found that ADM expression was increased in RCC, which promoted hypoxia-inducible factor 1 (HIF-1) and VEGF expression [17]. However, in some other studies, researchers found that ADM directly promoted endothelial cell growth and survival through activation of MAPK/ERK downstream signaling pathways [18]. Under serum deprivation, ADM promotes DNA synthesis and cell proliferation in vascular smooth muscle cells via p42/p44 MAPK pathway activation [19]. Additionally, in some solid tumors, ADM can upregulate Bcl-2 levels via autocrine/paracrine effects [20]. Silencing of the ADM gene in HO8910 cells (ovarian carcinoma cell line) can decrease Bcl-2 and phospho-ERK (p-ERK) expression, which inhibits cell proliferation [21].

Therefore, based on these data, we speculated that ADM expression may be associated with sunitinib resistance in RCC. Accordingly, the aim of our study was to investigate the potential role of endogenous ADM in the growth of sunitinib-resistant RCC by evaluating the effects of ADM/ADM receptor antagonists *in vivo* and *in vitro*. We demonstrated that ADM plays a significant role in sunitinib-resistant RCC cell proliferation via the ADM-ERK/MAPK pathway, while an ADM receptor antagonist blocks ERK activation and inhibits RCC cell proliferation.

## RESULTS

### Elevated ADM expression levels were associated with the sunitinib-resistant phenotype in the animal model

We searched the Gene Expression Omnibus(GEO) database, identified the following 2 sets of microarray data regarding renal cell carcinoma resistance to RTK inhibitors, and included these data in our study: GSE76068 [22] and GSE64052 [23]. In GSE76068, researchers implanted 8 RCC patient-derived xenografts in mice and then treated these mice with sunitinib for approximately 4 weeks, at which time drug resistance developed. The xenografts were collected for transcriptome analysis. Via the GPL10558 platform, reanalysis of these data showed that the post-treatment level of ADM expression was 2.67-fold higher than the pre-treatment level in tumors that were responsive to sunitinib treatment (*P <* 0.01). However, when sunitinib resistance developed, the post-treatment level of ADM expression was only 1.31-fold higher than the pretreatment level (*P <* 0.01), which may be due to heterogeneity with respect to patient-derived tumor responsiveness to sunitinib (Dataset 1).

**Figure 1:**
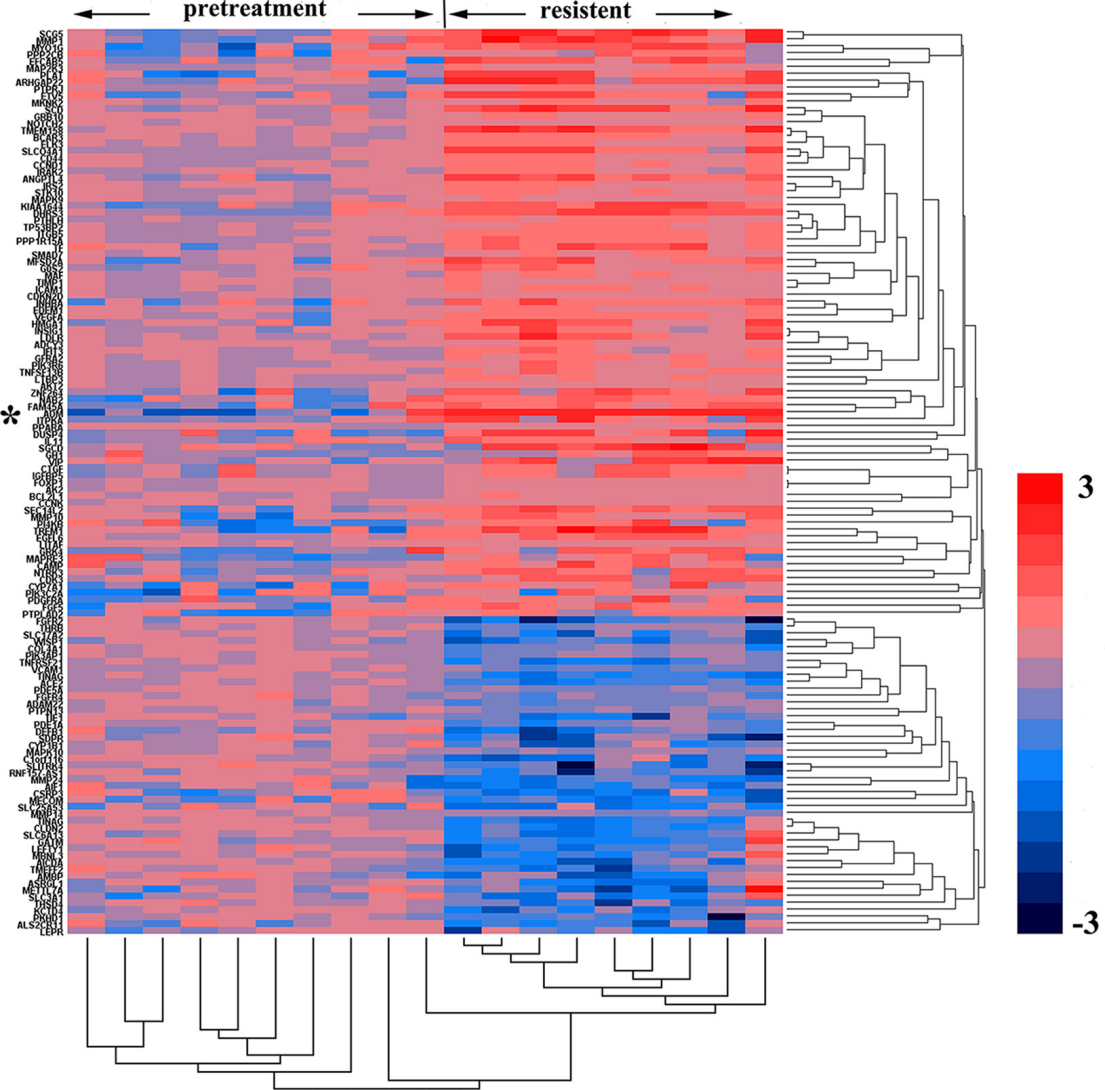
Genes and biological processes pertaining to acquired sunitinib resistance Heatmap representing gene expression changes in pretreated versus sunitinib-resistant murine 786-0 tumors. The columns represent the samples, and the rows represent the genes. Gene expression is shown via a pseudocolor scale, with red denoting high expression levels and blue denoting low expression levels.

In 2015, Zhang L et al. published their microarray data analysis of 786-0 cell xenografts that were resistant to sorafenib and sunitinib (GSE64052). We reanalyzed their raw data regarding gene expression (Figure 1) and noted that some genes were upregulated significantly when resistance developed, including those encoding VEGF, ADM, AKT2, CDKN2D, CD44, MAPK9, BCAR3, cAMP and genes responsible for cell survival, findings suggestive of the activation of cell proliferation (Dataset 2). The post-treatment level of ADM expression in sunitinib-resistant tumors was 3.98-fold higher than the pretreatment level (*P <* 0.01) and that the post-treatment level of MAPK9 expression was 7.76-fold higher than the pretreatment level (*P <* 0.01).

Therefore, we created an RCC mouse xenograft model to verify the expression of ADM in sunitinib-resistant tumors.

### ADM22-52(ADM receptor antagonist) inhibited sunitinib-resistant tumor growth

**Figure 2:**
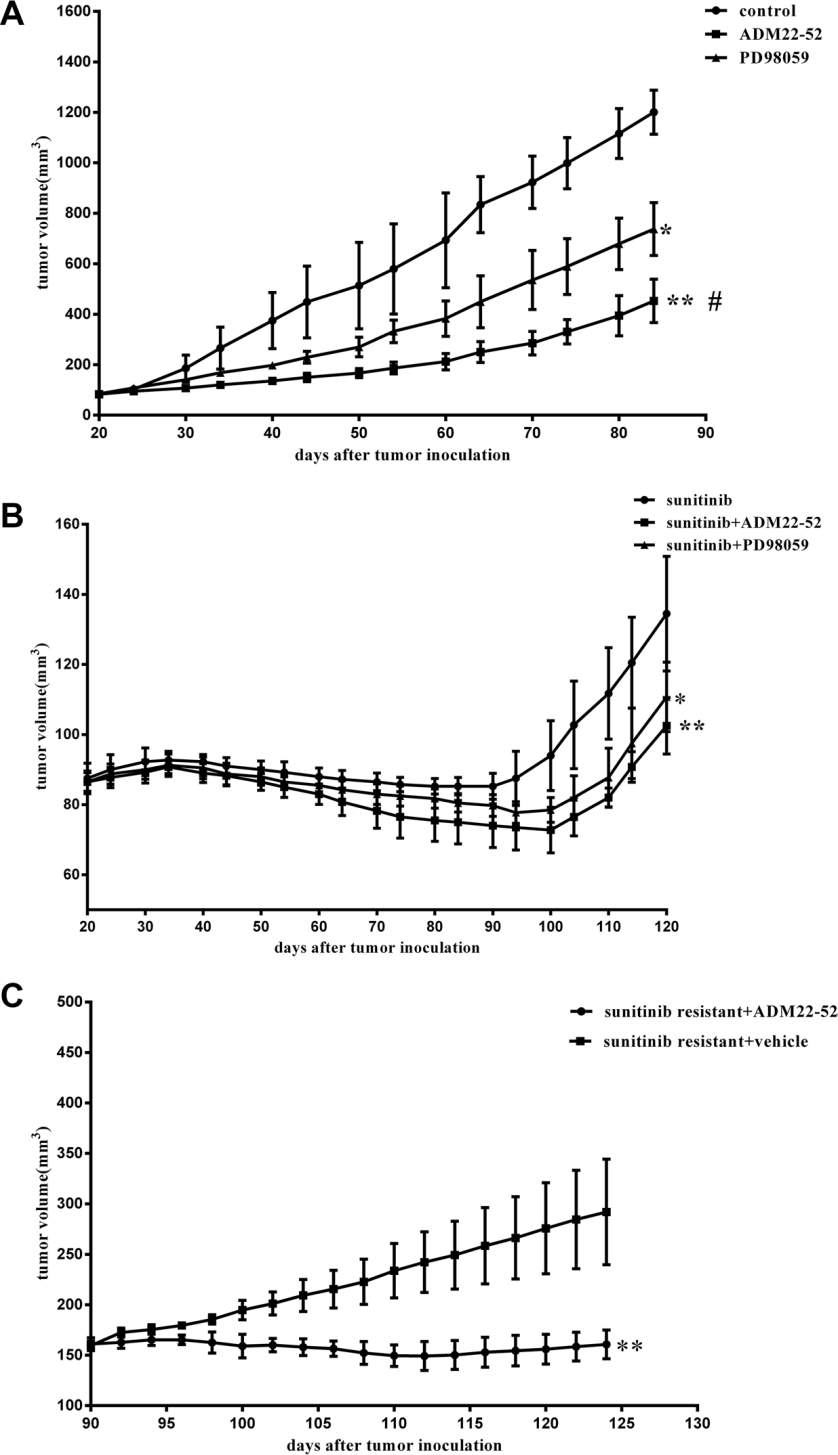
Effects of sunitinib, ADM22-52, PD98059 on the growth rates of mice RCC xenografts (**A**) and (**B**) Mice bearing tumors (4 mice/group) were treated with sunitinib, vehicle (control), ADM22-52, PD98059, sunitinib+ADM22-52, or sunitinib+PD98059 for an indicated number of days. Mean tumor volumes at specific time points are shown. Tumor volumes in the groups treated with sunitinib+ADM22-52 or sunitinib+PD98059 were compared to those in the groups treated with sunitinib. Tumor volumes in the groups treated with vehicle, ADM22-52, or PD98059 were compared to those in the groups treated with vehicle. (**C**) ADM22-52 inhibited sunitinib-resistant xenograft growth. 786-0 xenograft tumors were treated with sunitinib daily until phenotypic resistance developed. Then, the mice were randomly divided into two groups. One group was given sunitinib plus ADM22-52 (4 mice), and the others were given sunitinib plus vehicle (4 mice). ADM22-52 treatment began on day 90 and ended on day 130. Tumor volumes were monitored, and the results showed that sunitinib plus ADM22-52 treatment inhibited tumor growth compared with sunitinib plus vehicle treatment. Data are expressed as the mean ± SD (*p < 0.05; **p < 0.01. ^#^comparison between the ADM22-52 group and PD98059 group, *p* < 0.05).

Different groups of xenografts in mice were treated with sunitinib, ADM22-52, PD98059 (MAPK kinase inhibitor), sunitinib+ADM22-52, sunitinib+PD98059, or vehicle. Then, long-term tumor growth trends were investigated (Figure 2A and 2B). Compared to controls, both ADM22-52 and PD98059 suppressed xenograft growth, but ADM22-52 facilitated greater growth suppression than PD98059 (*P <* 0.05). Furthermore, compared to treatment with sunitinib alone, treatment with sunitinib+ADM22-52 or PD98059 resulted in significantly slower tumor growth. Therefore, we concluded that anti-tumor effects in tumors treated with sunitinib in combination with ADM22-52 or PD98059 were superior to sunitinib only, and we also hypothesized that tumor growth occurring independently of sunitinib treatment may be mediated by upregulation of ADM and activation of the ERK/MAPK pathway.

**Figure 3:**
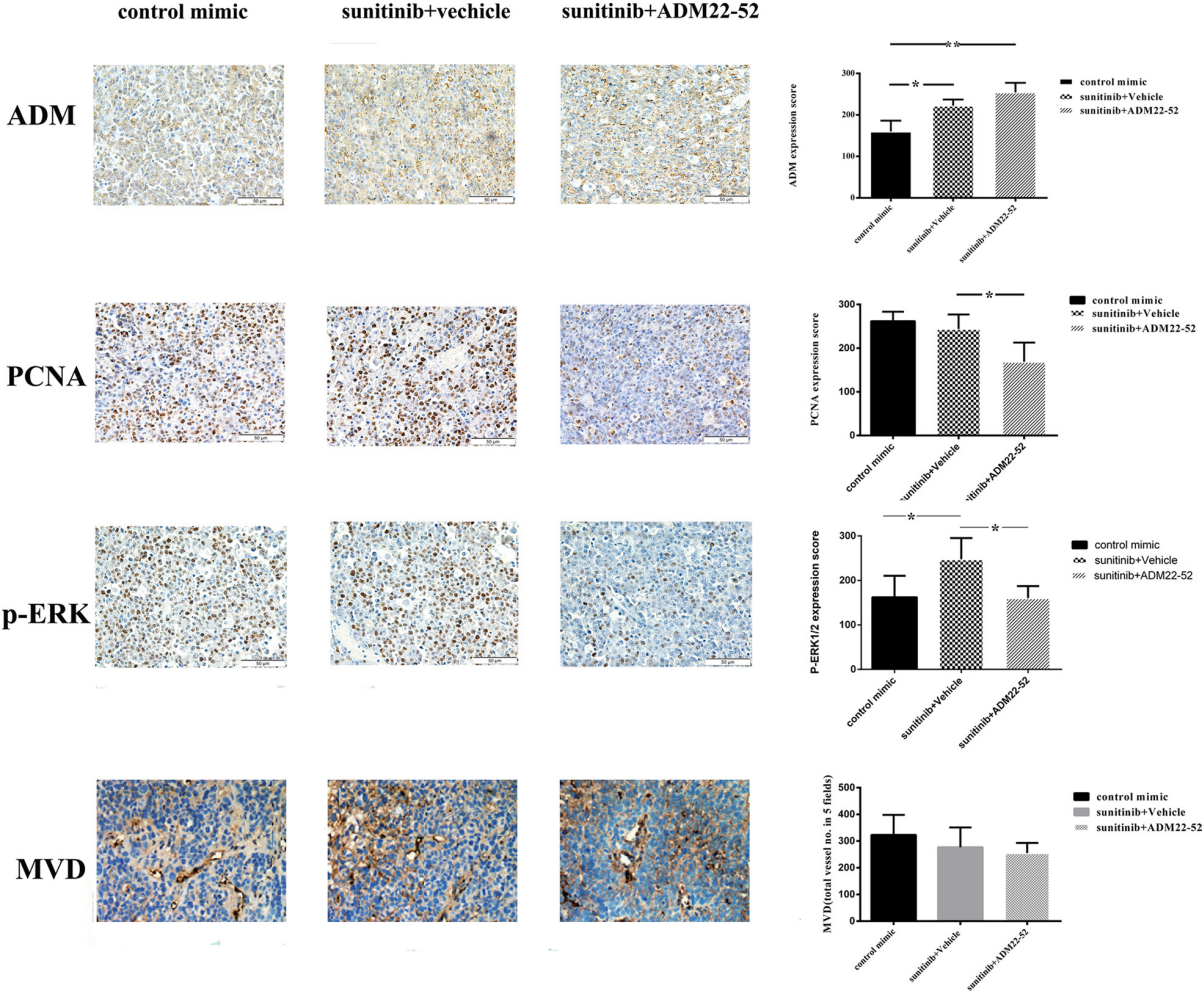
Elevated ADM, PCNA and p-ERK1/2 expression levels were noted in mice RCC xenografts that were resistant to anti-angiogenesis agents (sunitinib) Quantitative immunohistochemistry analysis and representative microscopic fields of ADM, PCNA, p-ERK1/2 staining and MVD (× 200). Columns, data are expressed as the mean ± SD, generated as described in the Materials and methods (*p < 0.05; **p < 0.01).

In the other *vivo* experiments, all 786-0 xenografts initially responded to treatment with sunitinib but developed resistance to therapy within 4 weeks. Subsequently, these mice were randomly divided into two groups: one group received sunitinib plus ADM22-52, and the other group received sunitinib plus vehicle. As shown in Figure 2C, sunitinib-resistant tumors began to significantly respond to treatment with the addition of ADM22-52 (*P <* 0.05). This phenomenon may be attributed to ADM22-52-mediated inhibition of the pathway regulated by ADM, which facilitates 786–0 cell survival independently of the VRGFR. Using IHC staining (Figure 3), we found that ADM expression was significantly increased in sunitinib-resistant tumors compared to untreated tumors (*P <* 0.05), accompanied by increased phospho-ERK1/2 expression (*P <* 0.05). Moreover, ADM expression was positively correlated with that of phospho-ERK1/2 in sunitinib+vehicle group (*P <* 0.05). PCNA is a biomarker of cell proliferation, and sequential administration of ADM22-52 after sunitinib resistance development significantly decreased PCNA (*P <* 0.05) and phospho-ERK1/2 expression (*P <* 0.05), compared with the sunitinib+vehicle group. In addition, we evaluated MVD levels via CD31 staining and found that ADM22-52 failed to decrease MVD levels in sunitinib-resistant tumors. In sunitinib-resistant tumors that were treated with sunitinib+vehicle, ADM expression was positively correlated with PCNA expression (*P <* 0.05), whereas ADM expression was not correlated with MVD expression (*P <* 0.05). Therefore, we concluded that ADM promotes growth of sunitinib-resistant tumors and that ADM receptor antagonist (ADM22-52) inhibits sunitinib-resistant tumor growth via a pathway other than the neo-angiogenesis.

### Effect of ADM on cell proliferation

**Figure 4:**
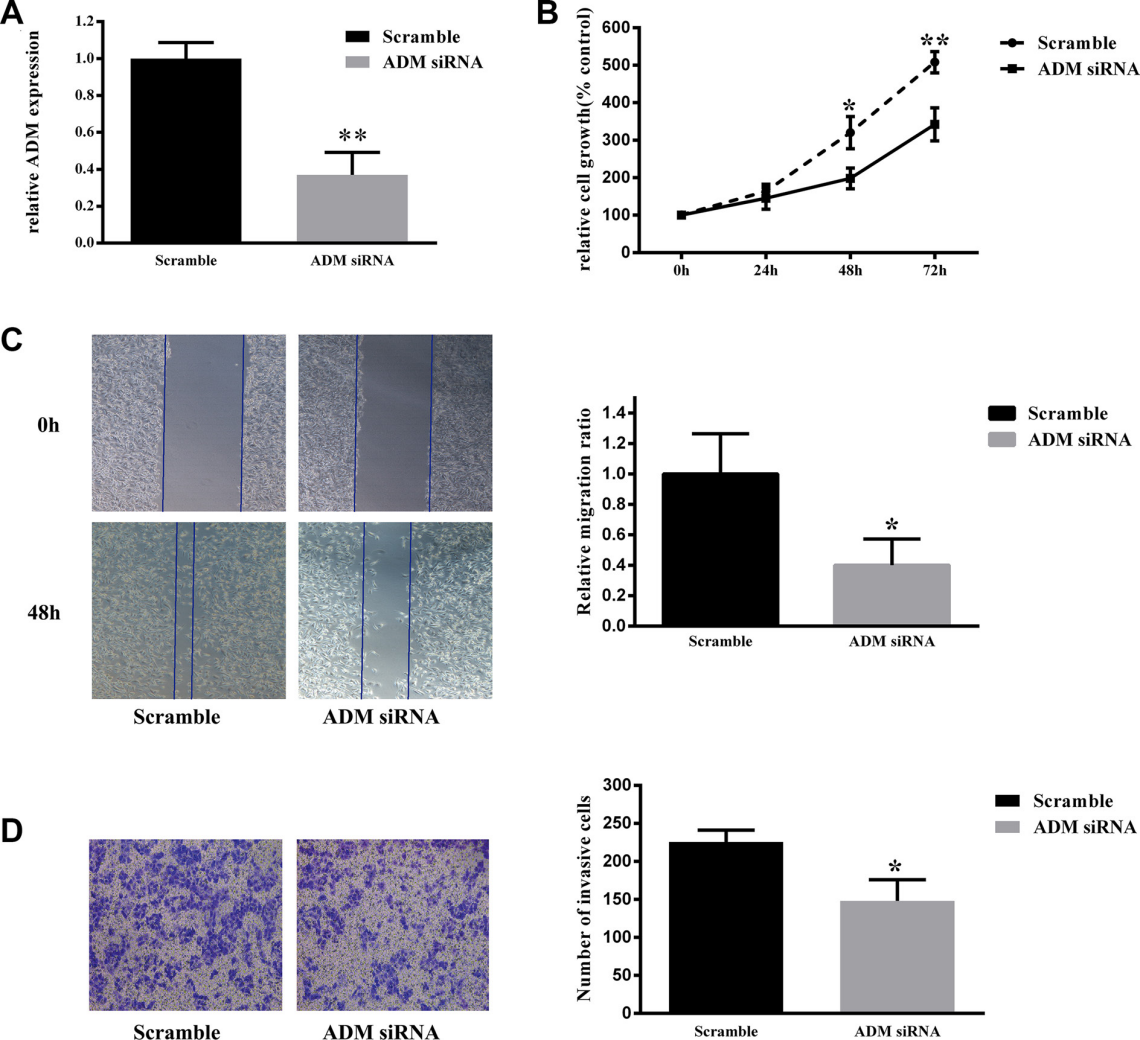
Down-regulation of ADM inhibited 786-0 cell proliferation, migration and invasion (**A**) Knockdown of ADM expression using siRNA decreased ADM expression. RNA was extracted from 786-0 cells transfected with ADM siRNA or scrambled control siRNA control for 3 days and analyzed via qRT-PCR. ADM mRNA levels were used for comparison. (**B**) Cell viability assay showed that ADM knockdown resulted in a significantly lower cell proliferation rate in 786-0 cells than in negative control cells. (**C**) Wound healing assay showed that ADM knockdown resulted in significantly lower migration capacity in 786-0 cells than in negative control cells. (**D**) Transwell invasion assay showed that ADM knockdown resulted in significantly lower invasion capacity in 786-0 cells than in negative control cells. The results are expressed as the mean ± SD for three replicate determinations. *P < 0.05, **p < 0.01.

After 786-0 cells were transfected with ADM siRNA, the expression level of ADM was significantly decreased compared with that of the negative control group (*P <* 0.01, Figure 4A). A cell viability assay showed that knockdown of ADM significantly inhibited 786-0 cell proliferation compared with the negative control group (*P <* 0.05, Figure 4B), which indicated that knockdown of ADM expression had an inhibitory effect on the proliferation of renal cancer cells.

### Effect of ADM on cell migration and invasion

A wound healing assay showed that the migration rate of 786-0 cells significantly decreased after transfection with ADM siRNA compared to that of negative control cells (*P <* 0.05, Figure 4C). Moreover, transwell invasion assay indicated that the invasion ability of 786-0 cells was significantly decreased via ADM knockdown compared to that of the negative control group (*P <* 0.05, Figure 4C). Therefore, we concluded that down-regulation of ADM restricted the migration and invasion ability of 786-0 cells *in vitro*.

### ADM expression increased in sunitinib-resistant RCC cells

**Figure 5:**
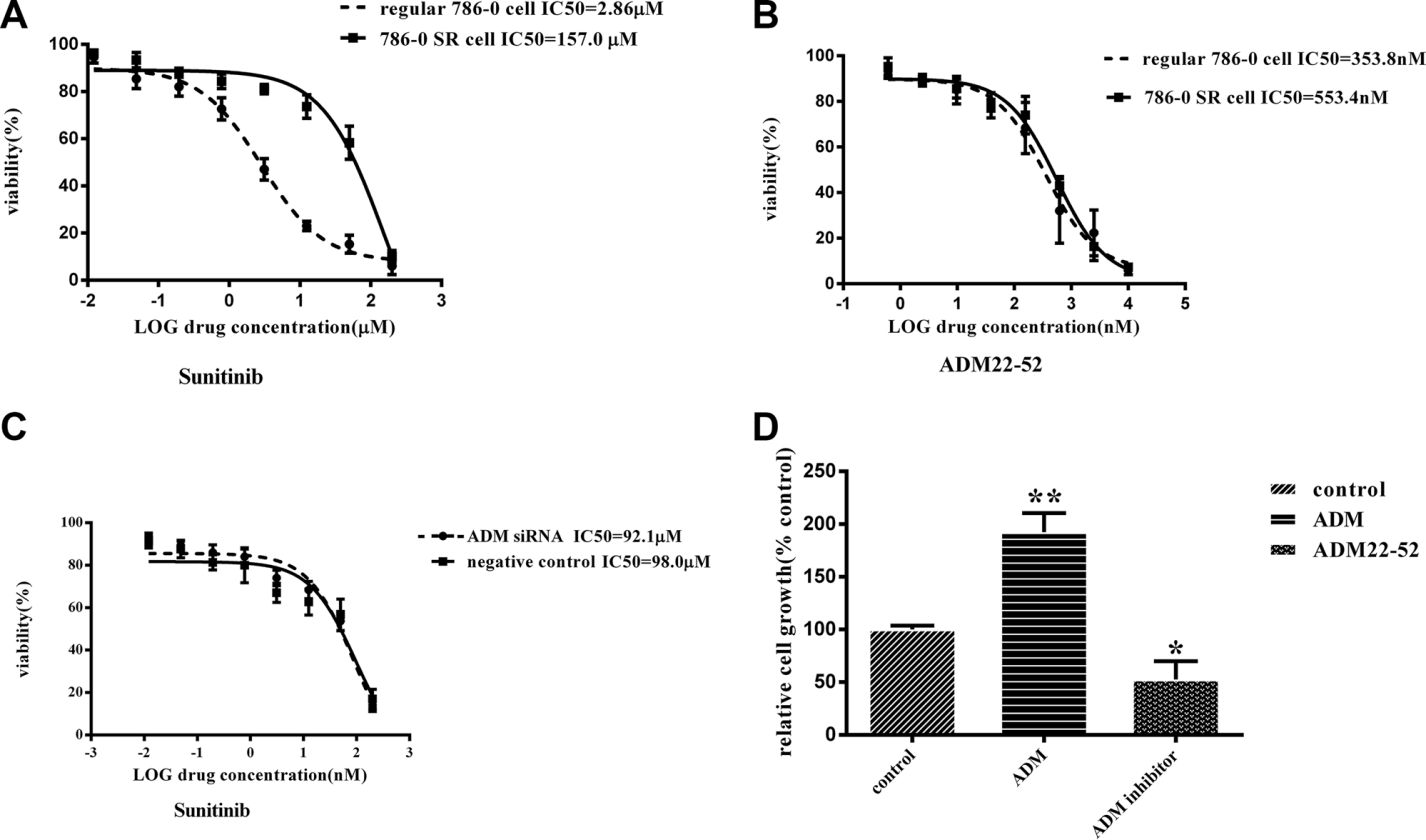
The effects of treatment with sunitinib or ADM22-52 on 786-0 cell growth *in vitro* (**A**) Regular 786-0 cells and 786-0SR cells were treated with the indicated doses of sunitinib(concentration ranged from 200 μM to 0.012 μM). (**B**) Regular 786-0 cells and 786-0SR cells were treated with the indicated doses of ADM22-52(concentration ranged from 200 μM to 0.012 μM). (**C**) 786-0SR cells transfected with ADM siRNA or its scrambled control, and then were treated with the indicated doses of sunitinib(concentration ranged from 200 μM to 0.012 μM). (**D**) The effects of ADM (10^−7^M) and ADM22-52(10^−6^M) on regular 786-0 cell growth *in vitro*. After 72 h of incubation, cell growth was determined in triplicate for all of the above experiments. Bars, mean ± SD. (*P < 0.05; **P < 0.01).

We cultured regular 786-0 cells and 786-0 sunitinib-resistant (SR) cells derived from sunitinib-resistant xenografts *in vitro* and then treated these cells with sunitinib and ADM22-52 respectively to evaluate their IC50 values. The curves showed that the IC50 value of sunitinib in 7860SR cells was 157 μM, which indicated that these cells were resistant to sunitinib. In contrast, the IC50 value of sunitinib in regular 786-0 cells was 2.86 μM, in Figure 5A (*P <* 0.01). However, the IC50s of ADM22-52 were similar in regular 786-0 cells and 786-0SR cells (353.8 vs. 553.4 nM, Figure 5B), indicating that the drug was effective against both cell types. In addition, the results showed that IC50 of sunitinib were 92.1 μM and 98.0 μM respectively, in 786-0SR cells with ADM knockdown and negative control, which indicated that knocked-down ADM expression cannot reverse the sunitinib sensitivity (Figure 5C).

**Figure 6:**
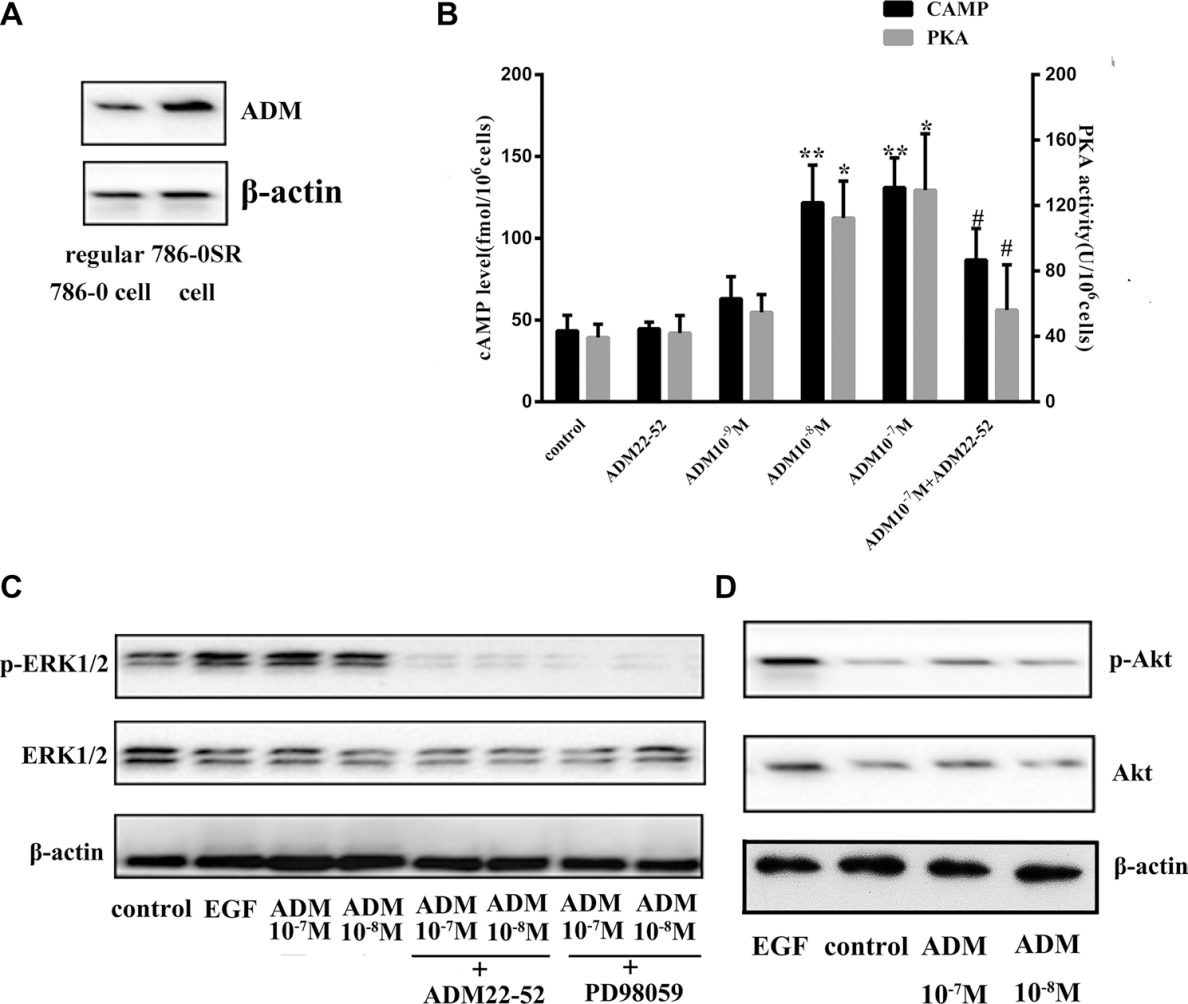
The ERK/MAPK pathway was induced by ADM in 786-0 cells (**A**) ADM expression was increased in 786-0SR cells. (**B**) Effects of ADM on cAMP levels and PKA activity. ADM induced cAMP formation and increased PKA activity in a dose-dependent manner in cultured 786-0 cells compared to the control group. Cells were treated with ADM at concentrations of 10^−7^-10^−9^ M or ADM22-52 (10^−6^ M) for 2 hours. Bars (mean ± SD) represent three independent experiments (*P < 0.05; **P < 0.01, ^*#*^comparison between the ADM (10^−7^ M) group and ADM22-52 (10^−6^ M)+ADM (10^−7^ M) groups, *p* < 0.05). (**C**) Cells were treated with ADM (10^−7^–10^−8^ M) for 2 h, and then cell lysis proteins were immunoblotted for phospho-ERK1/2, ERK1/2 and β-actin expression. PD98059 and ADM22-52 inhibited ADM-induced phosphorylation of ERK. EGF (5 ng/ml) was used as positive control known to stimulate ERK1/2 phosphorylation. Pre-incubation of the cells with ADM22-52 (10^−6^ M) or PD98059 (10 mmol/l) for 30 min inhibited ADM-induced phosphorylation of ERK1/2. β-actin was used as a loading control. (**D**) The intracellular signaling pathway associated with Akt expression in 786-0 cells treated with/without ADM.

We investigated 786-0 cell proliferation after treatment with ADM or ADM22-52, and the results showed that ADM significantly promoted cell proliferation (*P <* 0.01), while ADM22-52 significantly inhibited cell proliferation (*P <* 0.05) compared to the control group (Figure 5D). To determine if sunitinib can increase ADM expression, proteins were extracted from 7860SR cells and subjected to western blotting, and the data showed that ADM expression was much higher in these cells than in regular 786-0 cells (Figure 6A).

### ADM upregulated the ERK/MAPK pathway

We investigated whether ADM upregulated cyclic adenosine monophosphate (cAMP) and activated its downstream pathway protein kinase A (PKA). 786-0 cells were treated with ADM (10^−7−^10^−9^ M) or ADM22-52 (10^−6^ M) *in vitro*, and the results showed that cAMP levels and PKA activity both increased significantly in a dose-dependent manner in response to ADM treatment at concentrations of 10^−7^ M and 10^−8^ M compared to the control group (*P <* 0.05). In contrast, neither cAMP nor PKA upregulation was observed in cells treated with ADM22-52. In cells that were pre-incubated with ADM22-52, ADM failed to increase cAMP and PKA levels (Figure 6B). In addition, because 10^−9^ M ADM failed to increase cAMP levels, we treated 786-0 cells with ADM at concentrations of 10^−7^ M and 10^−8^ M to determine whether ADM could upregulate ERK1/2 expression. Cells were treated with ADM22-52 (10^−6^ M), PD98059 (10 mmol/l) or vehicle for 30 minutes, and then different concentrations of ADM (10^−7^ and 10^−8^ M) were added for 2 hours. The results indicated that ADM treatment increased phospho-ERK1/2 levels and that there was no significant difference in phospho-ERK1/2 levels following treatment with different concentrations ADM. However, phospho-ERK1/2 expression decreased when the ADM-ERK pathway was blocked by ADM22-52 or PD98059 (Figure 6C). In addition, we investigated Akt levels in 786-0 cells that were treated with ADM, but the data showed that ADM failed to increased phospho-Akt levels (Figure 6D).

### Effect of sunitinib+ADM22-52 on 786-0 cells

**Figure 7:**
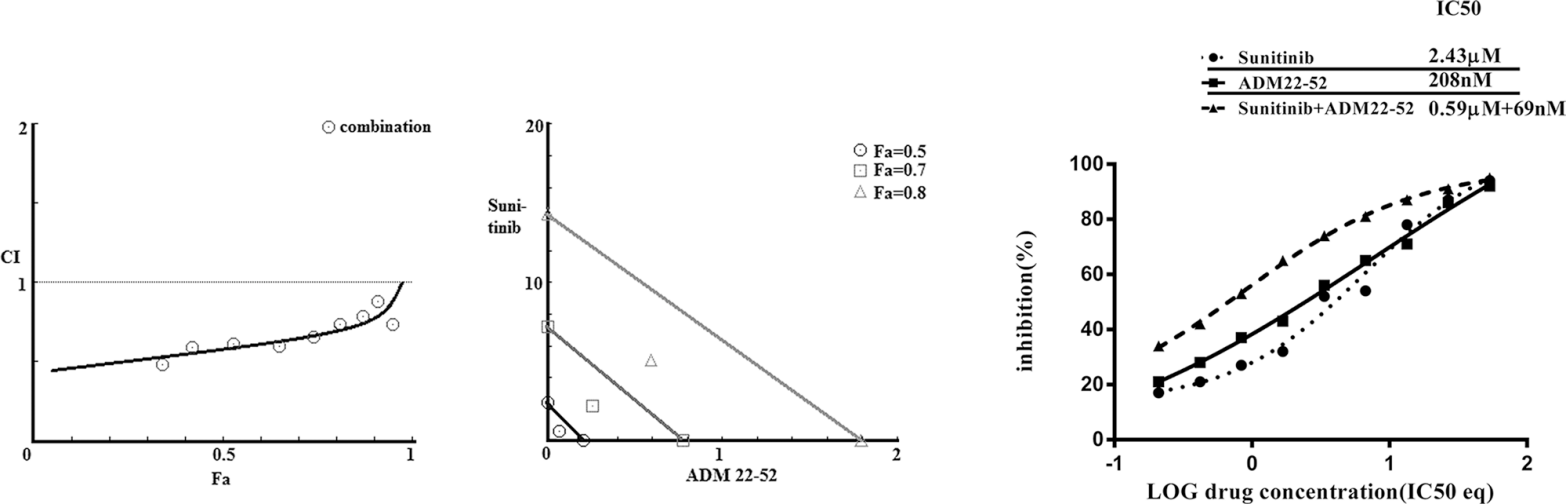
Combination treatment with sunitinib and ADM22-52 in 786-0 cells Left: Combination index (CI) analysis. Data for the combined treatment indicated that the curve was below 1, which was indicative of synergy. Middle: Isobologram analysis. At Fa = 0.5, 0.7 and 0.8, all the data points for the combination treatment were below the additivity line, which was indicative of synergy. Right: Curve-shift analysis. The combined treatment curves were shifted to the left, which indicated that synergy was achieved via the combination of sunitinib and ADM22-52.

We investigated the combined effects of sunitinib and ADM22-52 on 786-0 cells. This combination exerted synergistic effects (combination index, CI = 0.58), and this finding was consistent with those of our isobolographic analysis, in which all data points pertaining to the combination of the above two agents were below the line of additivity, irrespective of the effect level (Figure 7). At Fa (fraction affected; inhibition of cancer cell proliferation) = 0.5, 208 nM ADM22-52 or 2.43 μM sunitinib was required to induce 50% cancer cell inhibition when each agent was used alone. However, when the two agents were used in combination, ADM22-52 and sunitinib concentrations of only 69 nM and 0.59 μM, respectively, were required to achieve 50% inhibition, dose reductions of 3 and 4 fold, respectively.

## DISCUSSION

Sunitinib is currently one of the standard treatments for advanced ccRCC. However, ccRCC resistance to sunitinib remains an oncologic and clinical challenge. Therefore, we searched the GEO database and found microarray data regarding gene expression in sunitinib-resistant RCC tumors. Reanalysis of these raw data showed that upregulation of ADM expression and its downstream pathway may play a role in tumor resurgence following VEGF inhibition. To confirm the effects of ADM in sunitinib-resistant RCC, we established a mouse xenograft model, and the results showed that the development of sunitinib resistance was accompanied by increased ADM expression. We also showed that inhibition of ADM attenuated sunitinib-resistant tumor growth. Moreover, *in vitro*, an ADM receptor antagonist suppressed 786-0 cell proliferation by blocking the ERK/MAPK pathway. Synergistic anti-tumor effects were observed when RCC cells were treated with sunitinib in combination with an ADM receptor antagonist.

Gene expression-related changes in cell proliferation and angiogenesis appeared to compensate for the inhibition of VEGF/VEGFR-mediated signaling, which may be a common mechanism underlying the development of cancer resistance to VEGF pathway inhibitors [24, 25]. The GSE64052 and GSE76068 microarray datasets indicated that ADM and ERK/MAPK were significantly upregulated, which contributed to sunitinib-resistant tumor growth. Our data also demonstrated that sequential treatment of sunitinib-resistant tumors with an ADM receptor antagonist can reduce tumor growth. ADM is expressed in a variety of malignant tumor tissues, exhibits pro-mitogenic and pro-angiogenic effects, and is essential for the tumor growth *in vivo* [20, 26–28]. Fujita Y and his team suggested that ADM can increase VEGF expression by inducing HIF-1 upregulation, which promotes angiogenesis [17]. However, in our study, the VEGFR was blocked by sunitinib, which prevented ADM from promoting angiogenesis via the VEGFR pathway. Moreover, the ADM receptor antagonist failed to decrease MVD levels in sunitinib-resistant tumors, suggesting that tumor growth was inhibited through a pathway that was not associated with angiogenesis. Based on our reanalysis of the microarray data, we determined that the cell proliferation-related pathway ERK1/2 was upregulated in sunitinib-resistant tumors. Increased expression of phosphorylated ERK1/2 has been noted in various cancers, which can induce cancer cell proliferation and cancer progression [29]. In this study, we found that 786-0 cell proliferation was inhibited *in vivo* and *in vitro* by an ADM receptor antagonist, accompanied by decreased expression of phospo-ERK1/2. ADM increased phospo-ERK1/2 expression in 786-0 cells, while the ERK/MAPK pathway could not be activated if cells were pretreated with an ADM receptor antagonist or MEK inhibitor. Therefore, we concluded that ADM promoted 786-0 cell proliferation via upregulation of the ERK/MAPK pathway. In addition, the combination of sunitinib and the ADM receptor antagonist was superior to sunitinib treatment alone or the combination of sunitinib and an MEK inhibitor. This finding may be attributed to the dual effects of anti-angiogenesis and anti-cell proliferation by ADM receptor antagonist.

In summary, we demonstrated that blocking the ADM receptor inhibited proliferation of sunitinib-resistant RCC cells via the ERK/MAPK pathway, supporting the hypothesis that ADM can exert autocrine/paracrine effects promoting cancer growth in RCC. ADM expression by cancer cells promotes tumor growth, so combination therapy with sunitinib and an ADM receptor antagonist may have important implications for RCC treatment.

## MATERIALS AND METHODS

### Microarray data analysis

Microarray data regarding renal cell carcinoma cells that are resistant to RTK inhibitors were obtained from GEO (http://www.ncbi.nlm.nih.gov/geo/) to identify datasets suitable for reanalysis. The following keywords were used: ‘renal cell carcinoma’, ‘resistant’, and ‘RTK inhibitor’. Only 2 microarray platforms, GSE76068 [22] (GPL10558, Illumina HumanHT-12 V4.0 expression beadchip) and GSE64052 (GPL570, Affymetrix HG-U133 Plus 2.0) [23], were included in our study. Next, we download the above datasets (CEL files) and prepared the server for data analysis with R statistical software (www.r-project.org), using the Bioconductor library [30]. Analysis of these two datasets, which included information regarding genes related to cell signaling pathways, cytokine expression, and cellular metabolism, was subsequently performed. Gene expression levels were quantified after analysis, and heat map based on gene expression results was generated.

### Cell culture

The indicated renal clear cell adenocarcinoma cell line (786-0) was purchased from the American Type Culture Collection (ATCC) and maintained in DMEM (Hyclone, Logan, Utah, USA) supplemented with 10% ultracentrifuged fetal bovine serum (FBS; Invitrogen, CA, USA), 1 mM sodium pyruvate (Invitrogen), penicillin (100 U/ml; Invitrogen) and streptomycin (10 mg/ml; Invitrogen) at 37°C in a humidified atmosphere of 5% CO2. The cell culture also included 0.05% trypsin and 0.02% ethylenediamine tetra-acetic acid (Hyclone).

Sunitinib-resistant 786-0 cells (786-0 SR) were derived from sunitinib-resistant 786-0 tumor xenografts. Briefly, the xenografts were established and treated with sunitinib until resistance developed. At the end of the experiments, the mice were sacrificed, and the tumors were collected. Subsequently, the tumor tissue was minced into small pieces (1 mm^3^), incubated in 12.5 ml of DMEM supplemented with 0.06% collagenase A (Sigma, MO, US, CAT. C0130) overnight and then incubated in 12.5 ml of 0.05% trypsin and 0.02% EDTA for 1 hour at 37°C with 5% CO2. The cell pellets were collected, washed with PBS, and then seeded in 10 cm culture dishes under standard cell culture conditions, using DMEM supplemented with 10% FBS and an antibiotic. The cells were cultured for future experiments.

### Mouse xenograft models and treatment

All animal experiments were approved by the Institutional Animal Care and Use Committee of Hebei Medical University. Male BALB/c nu/nu nude mice (8 weeks old) were purchased from Beijing HFK Bioscience Co., Ltd. (Beijing, China), and housed in our experimental animal facility (5 mice per cage) under standard laboratory conditions, with free access to food and water. Each mouse was given s.c. injections of 2 × 10^6^ 786-0 cells suspended in 100 μl of PBS and mixed with 100 μl of Matrigel (BD Biosciences, CA, USA) in the right flank region.

The tumors were measured with a dial-caliper, and their volumes were determined using the formula length × width × height × 0.5. Tumor volumes were measured every two days and were presented as the mean ± SD. When the tumor volumes reached approximately 80 mm^3^, the mice were randomly assigned to different treatment groups. Groups 1 and 2 were treated with sunitinib (oral gavage 40 mg/kg/day, Selleck Chemicals, TX, USA) until resistance developed, and then group 1 (*n* = 4) received treatment with sunitinib plus ADM22-52 (intraperitoneal injection 50 μg/day, Sigma, MO, USA, dissolved in PBS) [31, 32]. Group 2 (*n* = 4) was treated with sunitinib plus vehicle. Group 3 (*n* = 4) was treated with only sunitinib. Group 4 (*n* = 4) was treated with sunitinib and ADM22-52. Group 5 (*n* = 4) was treated with sunitinib and the MEK inhibitor PD98059 (2.5 mg/kg, intraperitoneal injection, Sigma, MO, USA, dissolved in DMSO) [33]. Group 6 (*n* = 4) was treated with ADM22-52 alone, and group 7 (*n* = 4) was treated with PD98059 alone. Group 8 (*n* = 4) was treated with vehicle (intraperitoneal injections of PBS).

To investigate sunitinib resistance, tumor growth ratios were determined by dividing tumor volumes measured at indicated time points by the tumor volume measured at the start of sunitinib treatment. Xenograft tumors that either did not respond to treatment or progressed on treatment after an initial response were considered phenotypically resistant. Xenograft tumors that showed volume increased < 25% or regression were considered sensitive. In contrast, tumors showing volume increased > 25% compared to their initial volumes or continued growth after long-term observation were considered resistant. Due to the time needed for drug treatment to affect tumor volumes, we determined sensitivity or resistance after three measurements [11].

At the end of the experiments, the mice were euthanatized, and their tumors were collected, fixed in 4% paraformaldehyde and paraffin-embedded. Then, 5 μm-thick sections were prepared for subsequent immunohistochemical staining and analysis.

### ADM22-52 siRNA transfection

786-0 cells were transfected with anti-ADM siRNA or a scrambled probe, which was used as a control (Santa Cruz, CA, USA), at a final concentration of 40 nM, using Lipofectamine 2000 (Invitrogen), in accordance with the manufacturer's instructions. Transfection efficiency was validated via real time qRT-PCR, which was used to measure ADM expression. Scrambled RNA were transfected into cells as negative control.

### RNA isolation and real time qRT-PCR

RNA was extracted using an RNeasy mini kit (Qiagen, CA, USA). CDNA was generated using a reverse transcription kit (SuperScript III First-Strand Synthesis System; Invitrogen). Gene expression was measured with SYBR Green-based reagent (SYBR GreenER qPCR SuperMix for iCycler; Invitrogen) on an ABI 7900 Real-time PCR System (Applied Biosystems, CA, USA), according to the manufacturer's protocol. The primers for ADM were as follows: F-5′-ATGAAGCTGGTTTCCGTCG-3′ and R-5′-GACATCCGCAGTTCCCTCTT'. The primers for β-actin, which was used as an internal control, were as follows: F-5′-GTCTGCCTTGGTAGTGGATAATG-3′ and R-5′-TCGAGGACGCCCTATCATGG-3′. The PCR reactions were run in triplicate; values were quantified with the corresponding standard curves. Relative quantification of gene expression was performed using the 2^−ΔΔCt^ method.

### Cell proliferation assay and drug IC50 determination

Regular 786-0 cells and 786-0SR cells were seeded in 96-well plates at a density of 5000 cells/well and allowed to attach overnight. Then, the cells were treated with different drugs, including ADM and ADM22-52, for 3 days. Cell viability was subsequently measured via WST-1 assay (Roche Diagnostics, IN, USA), according to the manufacturer's instructions.

In addition, 786-0 SR cells transfected with ADM siRNA were seeded in plates at a density of 5000 cells/well, and cell proliferation was measured at 24 h, 48 h and 72 h after incubation, using the WST-1 method.

Absorbance was read at 440 nm and 690 nm using a Multimode Plate Reader (Biotek, VT, USA). Data were used to generate drug effect or cell proliferation rate curves.

### Effect of sunitinib+ADM22-52 treatment on 786-0 cells

To test the combined effects of sunitinib and ADM22-52 on 786-0 cells, we calculated the efficacies of sunitinib, ADM22-52 and sunitinib+ADM22-52. We treated regular 786-0 cells as follows: group (1) was treated with doses of sunitinib that were 1/8-, 1/4-, 1/2-, 1-, 2-, 4- and 8-fold higher than the IC50 of sunitinib; group (2) was treated with doses of ADM22-52 that were 1/8-, 1/4-, 1/2-, 1-, 2-, 4- and 8-fold higher than the IC50 of ADM22-52; and group (3) was treated with the combination of sunitinib and ADM22-52 at a constant ratio (based on the IC50 ratio of two agents). All cells were treated for 3 days, and then cell viability was assessed via WST-1 assay.

A method introduced by Chou was applied to evaluate the synergy, additivity and antagonism of the above combination drug treatment. The CI values based on the linear regression analysis were calculated using CompuSyn software (ComboSyn Inc., NJ. USA), in accordance with the method developed by Chou et al., whereby hyperbolic and sigmoidal dose-effect curves are transformed into a linear form [34]. Based on a CI algorithm, a plot of CI values at different fraction-affected levels (Fas) can be calculated by computational simulation programs. Inputting the “dose (D) and effect (fa)” values for each drug alone and for their combinations allows CI values to be generated at different Fa levels, based on the CI algorithm. This is also known as a Fa-CI plot or a Chou-Talalay plot.

CI values were used to interpret drug combinations [35, 36], using the following scoring system: < 0.1, very strong synergy; 0.1–0.3, strong synergy; 0.3–0.7, synergy; 0.7–0.9, moderate to slight synergy; 1, nearly additive; 1.1–1.45, slight to moderate antagonism; 1.45–3.3, antagonism; and > 3.3, strong to very strong antagonism.

To describe the dose-dependent interactions of the two drugs, isobolograms at effect levels representing 50%, 70%, and 80% inhibition of cancer cell proliferation were created. Because treatment with each agent alone or the combination of two agents usually reached 50% cancer cell inhibition, the 50% isobologram allowed an actual comparison of monotherapy vs. combined therapy. The 70% and 80% isobolograms were used to illustrate the utility of the combination at high effect levels that have practical implications in oncology [37]. In each of these isobolograms, additivity was determined by extrapolating the dose requirements for each drug in combination, based on the dose requirements for each drug alone. Data points above or below the line of additivity indicated antagonism or synergy, respectively. The drug concentrations required for combination therapy were compared to those required for each agent alone to achieve a particular effect and expressed as a fold change.

Drug-effect-shift analysis was also performed. We created drug-effect curves for inhibitors used alone and in combination. This conversion of drug concentrations into IC50 equivalents allowed direct comparisons between the dose-response curves for individual agents and those for their combinations. The IC50s of each inhibitor and their combinations were compared. Synergy was noted when the IC50 equivalents of a particular combination that were needed to achieve a given effect were lower than those of each agent alone [38].

### Wound healing assay

To assess cell migration, 786-0 cells transfected with ADM siRNA or negative control were seeded in 12-well plates and cultured to confluence. Subsequently, wounds were inflicted using a sterile pipette tip, debris was removed with PBS, and then the cells were allowed to continue growing in culture. The speed of wound closure was monitored and photographed at 0 and 48 hours. Three independent experiments were performed.

### Transwell invasion assay

Cell invasion assays (Invitrogen) were performed in a 24-well transwell chamber, which was precoated with 100 μg of Matrigel. 786-0 cells transfected with ADM siRNA or negative control were collected and re-suspended in serum free medium at a concentration of 1 × 10^5^ cells/ml. Cells in suspension (200 μl) were added to the upper chamber, and the bottom chamber was filled with 500 μl of culture medium containing 10% FBS. After incubation for 24 hours, the cells that passed through the filter were fixed and stained using 0.1% crystal violet. The numbers of invading cells were counted in five randomly selected fields under a microscope (Olympus).

### Enzyme-linked immunosorbent assay (ELISA)

786-0 cells (1×10^5^) that were treated with ADM (10^−7^−10^−9^ M, dissolved in PBS, Abcam, ab69116) or ADM22-52 (10^−6^ M) were collected and lysed for cAMP level and PKA activity measurements via ELISA (cAMP kit: Fisher Scientific, San Francisco, CA, USA. PKA kit: Calbiochem, San Diego, CA, USA), according to the manufacturer's instructions. Color intensity was measured at 405 nm.

### Western blotting

After 786-0 cells were pretreated with different drugs, including an ADM receptor antagonist (ADM22-52, 10^−6^ M), PD98059 (10 mmol/l), epidermal growth factor (EGF, 5 ng/ml), or vehicle for 30 minutes, all the cells were treated with ADM (10^−7^ M, 10^−8^ M) for 2 hours. Then, proteins were extracted from the cell pellets and solubilized in RIPA buffer cocktail with protease inhibitors (Santa Cruz). After protein concentrations were determined, equal amounts of protein (20 μg/lane) were separated on 8%–10% SDS-polyacrylamide gels and then transferred to nitrocellulose membranes, using standard electroblotting procedures. The membranes were blocked with 5% non-fat milk in Tris-Cl-buffered saline (TBS-T, 0.1% Tween-20) at room temperature for 2 hours and then incubated with primary antibodies (1:3000) to p44/42 MAPK (Erk1/2) (rabbit monoclonal, Cell Signaling Technology, MA, USA, cat. #4695), p-p44/42 MAPK (Erk1/2) (rabbit monoclonal, Cell Signaling Technology, cat. #9101), ADM (rabbit polyclonal, Abcam, ab69117), and β-actin (mouse monoclonal, Santa Cruz, sc-47778) at 4°C overnight. The immunoblots were washed with TBS-T, incubated with an anti-mouse or anti-rabbit (1:5000, Santa Cruz) secondary antibody at room temperature for 1 hour, and subsequently processed for enhanced chemiluminescence (ECL) detection, using Super Signal Substrate (Pierce, IL, USA). Signals were detected via a chemiluminescence detection system (Bio-Rad Laboratories, CA, USA).

### Immunohistochemical staining (IHC)

Sections (5 μm) on glass slides were deparaffinized, rehydrated and then subjected to endogenous peroxidase blockage in 3% H2O2 and antigen retrieval in boiling 10% citrate buffer. Slides were blocked with goat serum and then incubated with monoclonal antibodies (1:200 dilution) against ADM (rabbit polyclonal, Abcam, ab69117), PCNA (mouse monoclonal, Santa Cruz, SC-56), CD31 (mouse monoclonal, Thermo Fisher, 37-0700, CA, US) and p-p44/42 MAPK (rabbit monoclonal, Cell Signaling Technology cat. #9101) overnight at 4°C. The slides were then incubated with a horseradish peroxidase-labeled dextran polymer coupled to an anti-mouse or anti-rabbit (Abcam, ab93705) antibody for 30 min at room temperature after 3 washes in PBS buffer. Finally, the slides were developed with diaminobenzidine for 4 min and counterstained with hematoxylin after 3 washes in PBS. Staining specificity was confirmed by processing sections from the same paraffin block and omitting the primary antibody as a negative control. As a positive control, we performed reactions with tissue sections specified by the manuals of the antibody providers. Nuclear or cytoplasmic staining that was clearly distinguishable from the background was considered positive.

The slides were reviewed twice by 3 blinded investigators, using a Nikon E-400 microscope. To investigate expression levels, at least 500 epithelial cells were evaluated per area of tissue showing positive immunoreactivity. The staining level was defined as the percentage of cells with 0-no, 1-weak, 2-moderate, or 3-intense staining by visual inspection under 400 × magnification, and the staining score was calculated using an overall H score with a range of 0–300. Target protein expression was graded semiquantitatively according to the staining score results, and the mean values were used for statistical analysis. In addition, the slides were stained with CD31 specific for MVD. MVD levels were determined based on the total number of vessels in the five fields showing the highest vascular density [39].

### Statistics

Data are expressed as the mean ± SD of three independent experiments. One-way ANOVA or Fisher's test was used for statistical analysis. Differences were considered significant at *p* values less than 0.05.
